# Effects of Wing–Tail Coupling on Aerodynamic Performance of Flapping-Wing Aircraft

**DOI:** 10.3390/biomimetics11060424

**Published:** 2026-06-15

**Authors:** Chao Wang, Longtian Zhang, Hao Liu, Kaicheng Yu, Jing Wu, Mingkang Zhu

**Affiliations:** 1 Department of Mechanical Engineering, Dongguan University of Technology, Dongguan 523808, China; zhanglt567@163.com (L.Z.); tianshen907@gmail.com (H.L.); yukaicheng5680@163.com (K.Y.); 2017045@dgut.edu.cn (J.W.); 2 Dongguan Key Laboratory of Intelligent Bionic Robot Technology and System, Dongguan 523808, China

**Keywords:** bird-inspired flapping-wing aircraft, aerodynamic characteristics, wing–tail aerodynamic coupling

## Abstract

To address the limited understanding of the aerodynamic characteristics of bird-inspired flapping-wing aircraft across different flight phases and the unclear flow field interaction mechanisms between the wings and tail, this study performs three-dimensional numerical simulations based on a self-developed prototype using ANSYS Fluent and the overset mesh method. The aerodynamic effects of key tail parameters under different flight conditions are quantitatively evaluated, and the mechanisms of bidirectional wing–tail aerodynamic coupling are investigated. The results show that tail twist has a negligible influence on instantaneous lift and thrust during level flight, with a maximum variation of only 0.2 N, but significantly affects the overall aerodynamic moments of the aircraft. When the tail twist angle increases from 15° to 20°, the pitching moment increases by 6%. In contrast, during climbing flight, the tail pitch angle has a pronounced effect on lift and thrust, and its aerodynamic influence depends strongly on the aircraft angle of attack. At an aircraft angle of attack of 15°, the difference between the maximum and minimum cycle-averaged pitching moments reaches 0.2 N·m. Further analysis of vorticity fields and pressure distributions confirms the existence of distinct wing–tail aerodynamic coupling. The tail not only directly modifies the aerodynamic forces and moments acting on the aircraft but also alters the wing-generated flow structures, while the wing wake simultaneously influences the aerodynamic effectiveness of the tail. This bidirectional wing–tail aerodynamic coupling plays a critical role in shaping the aerodynamic response of the aircraft under different flight conditions. These findings clarify the aerodynamic roles of key tail parameters and reveal the underlying flow field interaction mechanisms across different flight phases, providing a theoretical basis for motion-parameter optimization and precise attitude control of bird-inspired flapping-wing aircraft.

## 1. Introduction

Inspired by the remarkable flight performance of birds, bird-inspired flapping-wing aircraft are unmanned aircraft designed by mimicking avian flight dynamics, and have attracted widespread research interest over the years [1,2,3]. Compared with fixed-wing and rotary-wing unmanned aircraft, bird-inspired flapping-wing aircraft possess inherent advantages in concealment, maneuverability, low power consumption and long-endurance flight, and have thus emerged as one of the key research topics in bionic aviation. Nevertheless, existing flapping-wing aircraft still lag far behind real birds in terms of maneuvering performance and adaptability to complex flow fields under low-Reynolds-number conditions [4,5,6,7]. Reproducing the efficient flight regulation mechanisms of birds remains a major challenge in the bionic design of such aircraft.

To address this challenge, numerous studies have systematically explored the individual aerodynamic characteristics and control functions of wings and tails. The results indicate that wing kinematics (e.g., flapping frequency, angle of attack, spanwise twist angle and wing folding amplitude) and tail parameters (e.g., tail shape and posture) exert considerable influences on the lift-drag performance and attitude stability of the aircraft [8,9,10,11,12,13,14,15,16,17,18,19,20,21,22]. However, most of these studies regard wings and tails as independent aerodynamic and control surfaces [23,24], neglecting their dynamic coupling during flight. As a result, the aerodynamic interaction mechanisms between wings and tails throughout various flight phases remain poorly understood.

Further studies have demonstrated that the coordinated motion of wings and tails is one of the key factors enabling birds to achieve highly maneuverable and stable flight [25,26,27,28]. For instance, flow field observations of raptors during gliding reveal distinct tail vortices and wingtip vortices behind the tail and wingtips, which together form the characteristic vortex system of gliding birds. As an additional lifting surface separate to the wings, the tail generates tail vortices that not only compensate for insufficient lift produced by the wings but also realize real-time regulation of overall aerodynamic moments [29,30]. Behavioral observations of gliding birds also confirm that wing and tail movements tend to synchronize as flight velocity rises. Both wing and tail projected areas decrease as flight speed rises, along with obvious sweeping and retracting motions [31,32,33,34].

Implementing avian wing–tail coordination principles into bionic aircraft has greatly improved their flight stability [35,36,37], maneuverability [38,39,40] and aerodynamic efficiency [41,42,43]. Even so, relevant studies still have notable limitations. First, most current research on wing–tail coordination mainly concentrates on gliding instead of flapping flight, and the underlying aerodynamic principles differ significantly between the two states [44,45,46,47]. Second, the coupled aerodynamic effects of wing–tail coordination are hard to parameterize, and relevant variables are difficult to define. These obstacles prevent us from clarifying the flow mechanisms and quantitative control laws in different flight phases, leaving a lack of reliable theoretical guidance for the full-envelope flight control of FWAVs [48].

To address the above issues, this study builds on our previously developed FWAV prototype with a multi-degree-of-freedom tail structure [49]. A simplified three-dimensional model is established for numerical simulations using ANSYS Fluent 2022 R1, and the overset mesh method is employed to investigate flight attitudes during climbing and cruising.

This work quantifies the effects of wing-flapping-induced downwash distribution and vortex shedding characteristics, as well as wing–tail coupling, on the overall lift–drag performance and pitching moment under different flight attitudes. It also reveals how tail attitude adjustment regulates vehicle moment balance and clarifies the bidirectional coupling between the tail- and wing-induced downwash flow and vortex shedding.

The obtained results enrich the fundamental understanding of wing–tail aerodynamic coordination in flapping flight. This work provides theoretical references and quantitative data for the attitude coordination control and structural optimization of FWAVs across the full flight envelope.

## 2. Methods and Validation

### 2.1. Model Preprocessing and Flow Field Mesh Generation

**Figure 1:**
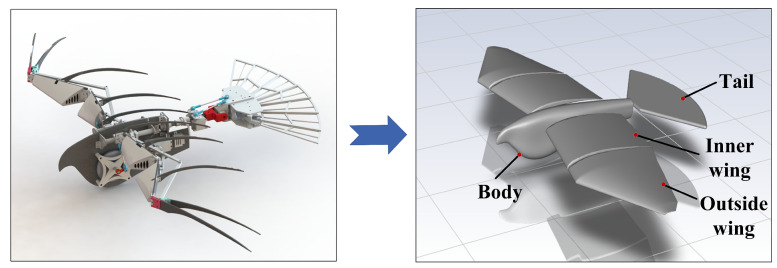
Three-dimensional simplified model of the bird-inspired flapping-wing air vehicle (FWAV).

The simulations in this study are performed in ANSYS Fluent. Prior to mesh generation, the three-dimensional simulation model of the air vehicle needs to be imported into Workbench Mesh. However, the actual modeling process of the flapping-wing air vehicle involves the design of multiple mechanisms, with a large number of parts and complex connection modes between various components. Therefore, to reduce the computational resources consumed by the numerical simulations, the three-dimensional model must be simplified before import. The simplified three-dimensional model of the bird-inspired flapping-wing air vehicle (FWAV) is shown in Figure 1.

As shown in Figure 1, the FWAV is divided into six regions. The flapping structure and the main frame in the fuselage region are simplified as the main body; the wing is divided into an inner wing and an outer wing; and the tail assembly is simplified as a sector-shaped thin plate. The basic parameters of the overall model are listed in Table 1.

**Figure 2:**
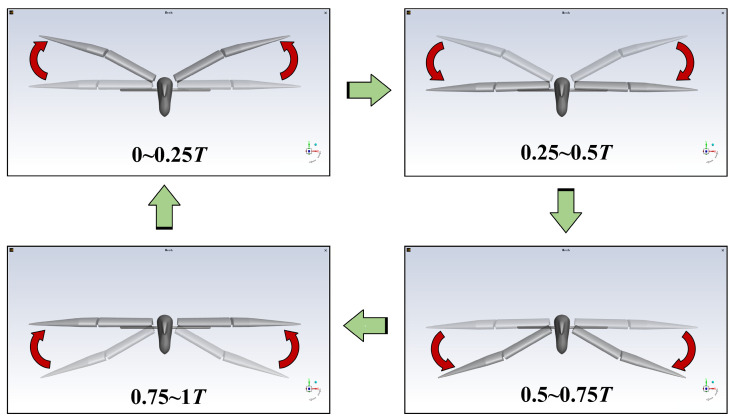
Schematic diagram of flapping wing motion decomposition within a single flapping cycle.

Meanwhile, the motion of each region can be controlled independently via the User-Defined Function (UDF), where the decomposition of the flapping wing motion within a single flapping cycle is shown in Figure 2. In the figure, the maximum flapping upward angle of the aircraft is 20°, and the maximum flapping downward angle is 38°.

**Figure 3:**
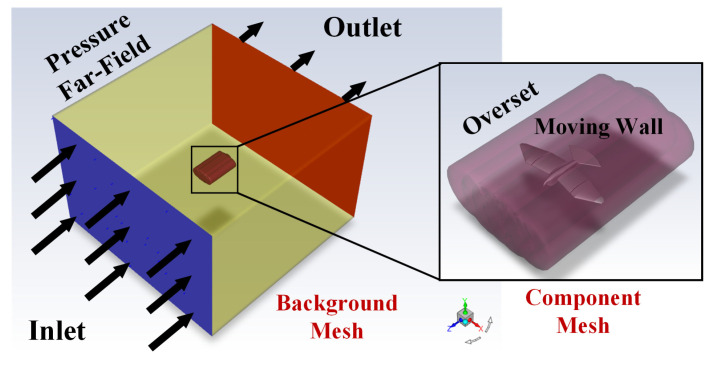
Computational domain model.

Furthermore, the overset grid method was adopted in this study for numerical simulations, which required region-wise independent mesh generation (background mesh and foreground mesh). The detailed computational domain model is illustrated in Figure 3, where the background mesh corresponds to the outermost rectangular flow domain, and each moving component of the flapping wing aircraft is enclosed within its corresponding cylindrical foreground mesh. The detailed dimensional parameters of the background mesh and each foreground mesh are summarized in Table 2.

Considering the Reynolds number range of 10^4^ to 10^5^ in this study, the shear stress transport (SST) *k*-*ω* turbulence model—the most widely used Reynolds-Averaged Navier–Stokes (RANS) model—was selected due to its superior near-wall treatment capability and high accuracy in predicting separated flows [50,51].

As illustrated in Figure 3, the blue region was specified as the velocity inlet with a free-stream velocity of 5 m/s and a turbulence intensity of 5%; the red region was defined as the pressure outlet with a relative static pressure of 0 Pa. The surfaces of all moving components of the aircraft were treated as no-slip moving walls, and the dimensionless wall distance *y*+ being less than 1 over the entire aerodynamic surface throughout the flapping cycle.

**Figure 4:**
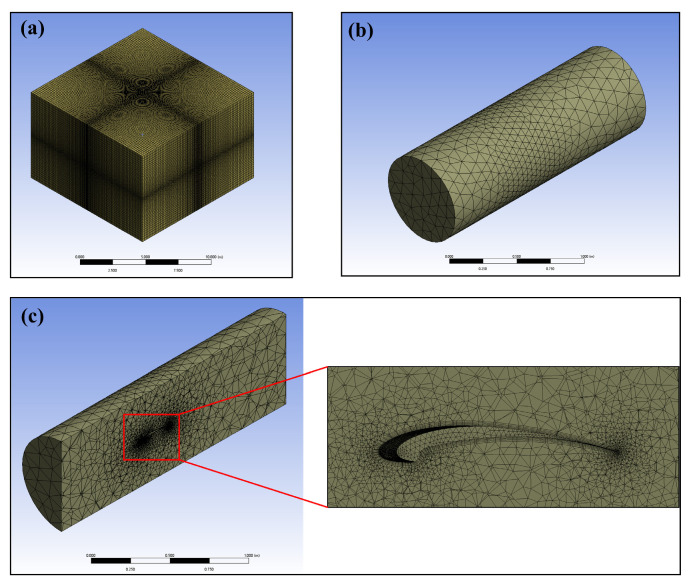
Schematic of partial computational domain meshing. (**a**) Schematic of background meshing. (**b**) Schematic of the right inner wing foreground meshing. (**c**) Schematic of boundary layer Inflation meshing on the inner wing surface.

Mesh generation was performed for the background mesh and each foreground mesh. Figure 4 shows a schematic of the partial grid, where Figure 4a,b display the schematic of the background mesh and the right inner wing foreground mesh, respectively. Figure 4c presents the schematic of the boundary layer inflation mesh on the inner wing surface.

### 2.2. Numerical Simulation Validation

**Figure 5:**
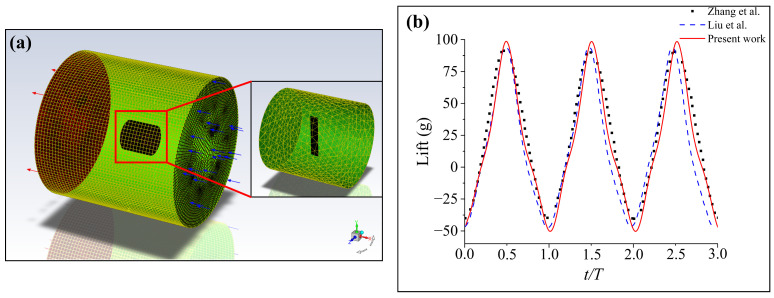
Mesh and simulation results for the NACA0012 lift force case. (**a**) Overset mesh. (**b**) Lift force comparison curves. To verify the accuracy of the CFD method, comparisons were made with the experimental data of Zhang et al. [52] and the CFD results of Liu et al. [53].

To verify the accuracy of the CFD method, comparisons were made with the experimental data of Zhang et al. [52] and the CFD results of Liu et al. [53]. A three-dimensional NACA 0012 airfoil consistent with the references was adopted as the standard motion model. The schematic of the computational domain grid is shown in Figure 5a, and the model parameters and motion parameters are listed in Table 3. By setting the same flow field environment and model motion parameters, the lift force comparison curves within three cycles are shown in Figure 5b. The residual type used in the simulations is scaled residuals, and the absolute criterion is adopted as the convergence criterion. The specific thresholds for the continuity equation, *x*/*y*/*z* momentum equations, k equation, and ε equation are all set to 10^−3^. An iteration step is considered converged when the scaled residual values of all governing equations with convergence checking enabled fall below the corresponding thresholds.

**Figure 6:**
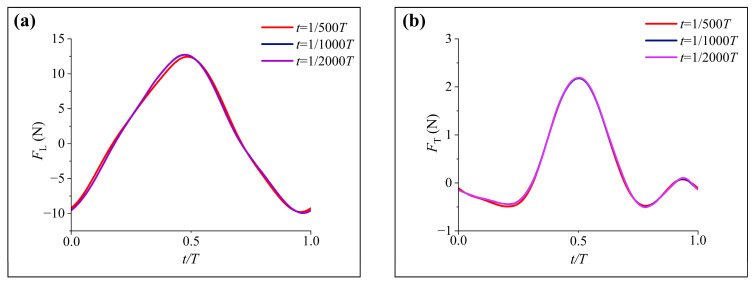
Comparison of lift and thrust curves for different time steps. (**a**) Instantaneous lift curves $F_{L}$ at different time steps. (**b**) Instantaneous thrust curves $F_{T}$ at different time steps.

**Figure 7:**
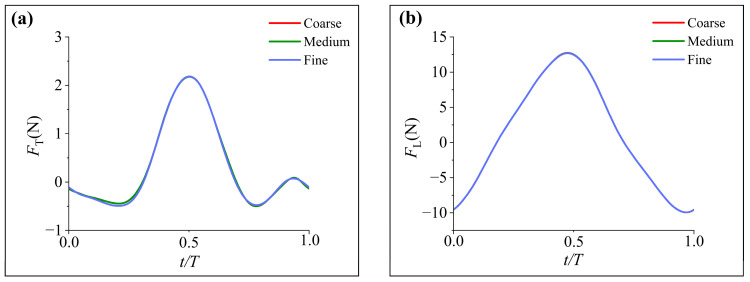
Comparison of lift and thrust curves for different mesh densities. (**a**) Instantaneous lift curves $F_{L}$ for different mesh densities. (**b**) Instantaneous thrust curves $F_{T}$ for different mesh densities.

Meanwhile, the simplified three-dimensional simulation model in this study was further introduced to perform grid independence verification and time-step independence verification. Both verifications were carried out under the working conditions listed in Table 4 for simulation analysis. By varying three levels of grid density (shown in Table 5) and three time-step sizes (1/500 *T*, 1/1000 *T*, 1/2000 *T*), the variations in lift force and thrust force curves within a single cycle under different grid densities or time steps for the same working condition were compared and analyzed. The results of time-step independence verification and mesh independence verification are shown in Figure 6 and Figure 7, respectively.

As shown in Figure 6, the instantaneous lift and thrust curves at time steps *t* = 1/500 *T* and *t* = 1/1000 *T* show nearly perfect overlap. Although the instantaneous lift and thrust at *t* = 1/2000 *T* deviate slightly from the two aforementioned cases, the discrepancy is negligible. To minimize computational resource consumption while guaranteeing sufficient numerical accuracy, a time step of *t* = 1/1000 *T* is selected for all subsequent simulations and analyses in this study.

The mesh independence verification was conducted at the time step of *t* = 1/1000 *T*. As shown in Figure 7, the instantaneous lift and thrust curves for the three mesh densities exhibit nearly perfect overlap. To minimize computational resource consumption while guaranteeing sufficient numerical accuracy, the medium-density mesh is adopted for all subsequent simulations and analyses in this study.

### 2.3. Kinematic Attitude Configuration in Level Flight Phase

**Figure 8:**
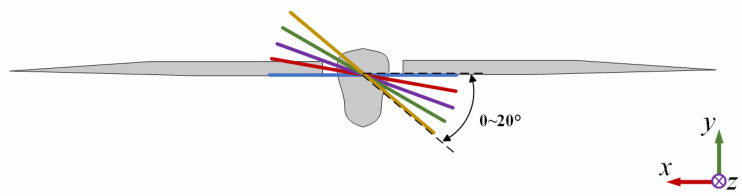
Schematic diagram of tail twist motion.

**Figure 9:**
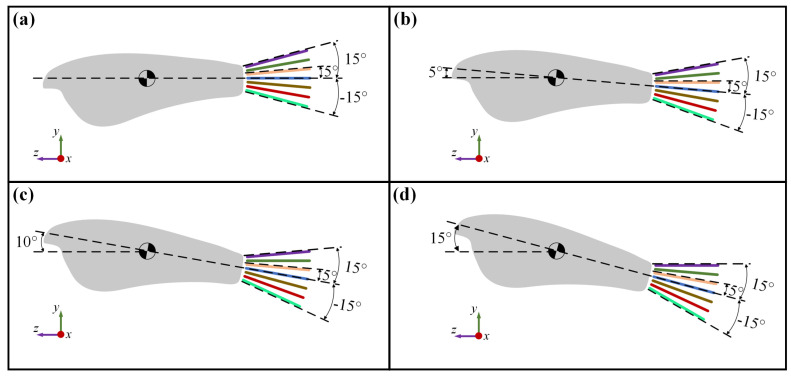
Schematic of aircraft attitudes for varying full-aircraft angles of attack and tail pitch angles. (**a**) Whole-aircraft angle of attack of 0°. (**b**) Whole-aircraft angle of attack of 5°. (**c**) Whole-aircraft angle of attack of 10°. (**d**) Whole-aircraft angle of attack of 15°.

As shown in Figure 8, the coordinate system is defined with its origin located at the center of gravity (CG) of the aircraft (as illustrated in Figure 9), where the positive directions of the pitching, rolling and yawing moments correspond to nose-down pitching, rightward rolling and leftward yawing of the entire aircraft, respectively. Five tail twist angles (0°, 5°, 10°, 15° and 20°) were set to investigate the aerodynamic performance of this flapping-wing aircraft, which achieves directional control solely through tail twist, during horizontal flight. The corresponding simulation conditions for kinematic parameters are summarized in Table 4.

### 2.4. Kinematic Attitude Configuration in Climb Phase

During the climb phase of the flapping-wing aircraft, the process typically involves simultaneous variations in both the full-aircraft angle of attack and the tail pitch angle. Accordingly, as shown in Figure 9, different full-aircraft angles of attack (0°, 5°, 10°, and 15°) and tail pitch angles (−15°, −10°, −5°, 0°, 5°, 10°, and 15°) were implemented as attitude variables in this study, with the corresponding kinematic parameter simulation conditions summarized in Table 6.

## 3. Results and Discussions

### 3.1. Effect of Tail Twist Angle During Level Flight Phase on Aerodynamics

**Figure 10:**
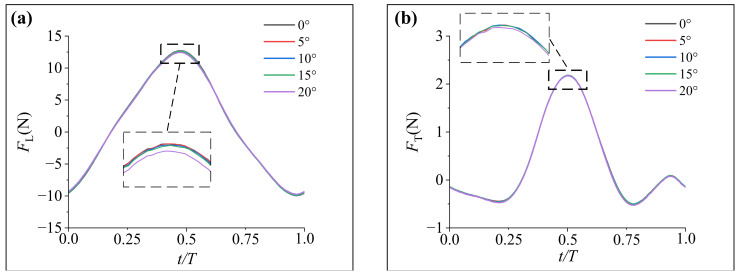
Comparison of instantaneous lift and thrust curves within a single flapping cycle for different tail twist angles. (**a**) Instantaneous lift results for different tail twist angles. (**b**) Instantaneous thrust results for different tail twist angles.

Simulation analysis was performed based on the above working conditions, and the obtained curves of the overall instantaneous lift and thrust of the full bird-inspired FWAV within a single flapping cycle under different tail twist angles $\theta_{T}$ are presented in Figure 10. The variation in the tail twist angle $\theta_{T}$ has a minor effect on the peak values of the full vehicle’s instantaneous lift and thrust. As can be seen from the local enlarged views of the wave peaks and troughs, for the instantaneous lift curves, the peak instantaneous lift values of the vehicle are almost coincident when the tail twist angle $\theta_{T}$ ranges from 0° to 15°, while a slight drop occurs in the peak instantaneous lift at the tail twist angle $\theta_{T}$ of 20°. This trend is also observed in both the peaks and troughs of the instantaneous thrust curves.

This minor change arises from the reduced projected area of the tail on the horizontal plane with increasing tail twist angle, which in turn weakens the tail’s lift generation during flight. The instantaneous lift and thrust data confirm that variations in tail twist angle $\theta_{T}$ have no direct impact on the magnitude of the full vehicle’s overall lift and thrust.

Beyond the variations in lift and thrust, tail twist in the level flight phase typically triggers attitude changes (e.g., rolling motion) of the bird-inspired flapping-wing aircraft. These attitude variations are directly reflected in the changes in the full vehicle’s overall moments. Accordingly, further analysis is required to investigate the impact of different tail twist angles $\theta_{T}$ on the full vehicle’s overall moments.

**Figure 11:**
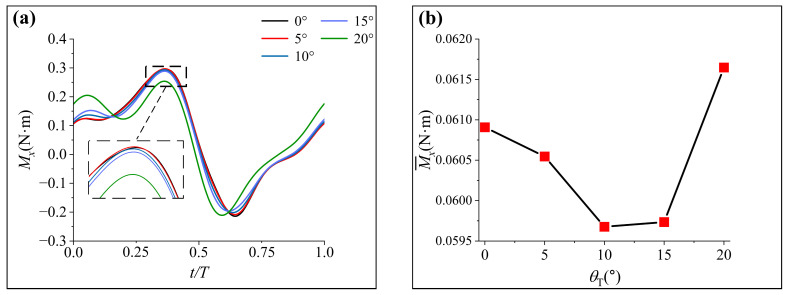
Instantaneous and cycle-averaged pitching moment results within a single flapping cycle. (**a**) Instantaneous pitching moment curve. (**b**) Cycle-averaged pitching moment line plot.

**Figure 12:**
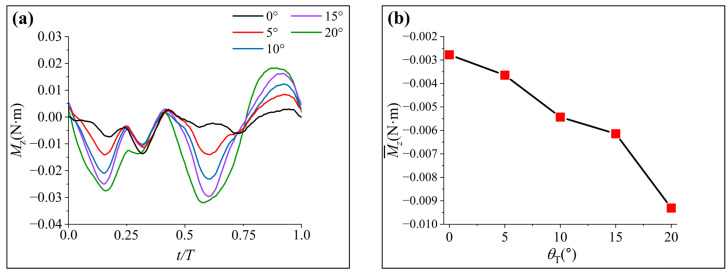
Instantaneous and cycle-averaged rolling moment results within a single flapping cycle. (**a**) Instantaneous rolling moment curve. (**b**) Cycle-averaged rolling moment line plot.

**Figure 13:**
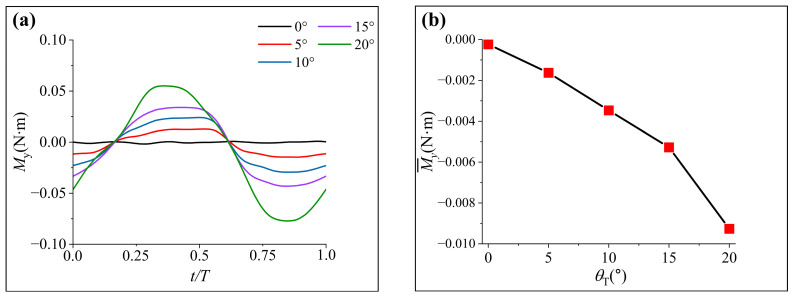
Instantaneous and cycle-averaged yawing moment results within a single flapping cycle. (**a**) Instantaneous yawing moment curve. (**b**) Cycle-averaged yawing moment line plot.

For varying tail twist angles, Figure 11, Figure 12 and Figure 13 present the comparison curves of the full vehicle’s instantaneous pitching ($M_{x}$), rolling ($M_{z}$) and yawing ($M_{y}$) moments within a single flapping cycle, alongside the line plots of the cycle-averaged counterparts over one flapping cycle, respectively.

As shown in Figure 11a, the instantaneous peak pitching moment rises with increasing tail twist angle $\theta_{T}$ over *t* = 0~0.25 *T*, with a pronounced increase observed at a twist angle of 20°. Conversely, over *t* = 0.25~0.5 *T*, the instantaneous peak pitching moment declines with rising tail twist angle, with a marked reduction at the 20° twist angle. As seen in Figure 11b, the cycle-averaged pitching moment $\bar{M_{x}}$ exhibits a decreasing trend as the twist angle increases from 0° to 10°. The variation trend of $\bar{M_{x}}$ levels off at a twist angle of 15°, before a sudden, rapid surge occurs at 20°. This demonstrates that tail twist induces changes in the overall angle of attack of the full bird-inspired FWAV. Moreover, as all cycle-averaged pitching moment $\bar{M_{x}}$ values are positive, when the tail twist angle $\theta_{T}$ increases progressively to a critical limit, the rapid rise in pitching moment may trigger a sharp reduction in the vehicle’s angle of attack, thereby resulting in variations in the vehicle’s flight altitude.

Furthermore, from the comparison of the curves of the instantaneous rolling moment $M_{z}$ and instantaneous yawing moment $M_{y}$ in Figure 12a and Figure 13a below, it can be seen that the instantaneous peak values of both the instantaneous rolling moment $M_{z}$ and instantaneous yawing moment $M_{y}$ increase with the rise in the tail twist angle. Meanwhile, combined with the line plots of the cycle-averaged rolling moment $\bar{M_{z}}$ and cycle-averaged yawing moment $\bar{M_{y}}$ in Figure 12b and Figure 13b, it can be observed that the magnitude of both cycle-averaged moments increases with the increase in the tail twist angle $\theta_{T}$. In addition, the direction of the rolling moment is opposite to the tail twist direction, and the fuselage yaw direction induced by the yawing moment is also opposite to the tail twist direction. This variation indicates that tail twist enables the bird-inspired flapping-wing aircraft to complete a steering maneuver opposite to the tail twist direction by simultaneously affecting the rolling moment and yawing moment, and the magnitude of the steering maneuver is positively correlated with the tail twist angle. This phenomenon is consistent with the conclusions obtained by Phan et al. [40] in their study on the cooperative tail motion during the gliding phase.

**Figure 14:**
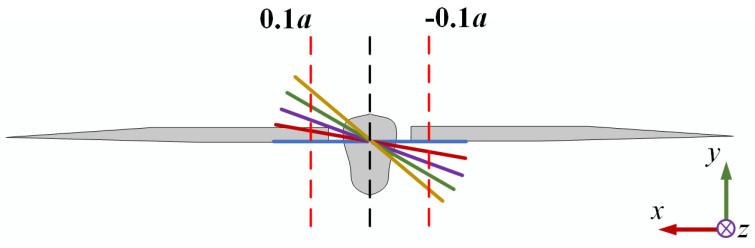
Schematic of left and right cross-section locations.

To further explore the mechanism by which tail twist angle $\theta_{T}$ variation modulates the flow field structure of the bird-inspired flapping-wing aircraft, we select the left and right cross-sections symmetric about the fuselage central axis for investigation, as presented in Figure 14. Each cross-section is located at a distance of 0.1a times the vehicle’s semi-span a from the fuselage central axis. The aerodynamic coupling between the periodic wing flapping and the tail is analyzed using the flow field vorticity and pressure contours of the two cross-sections.

**Figure 15:**
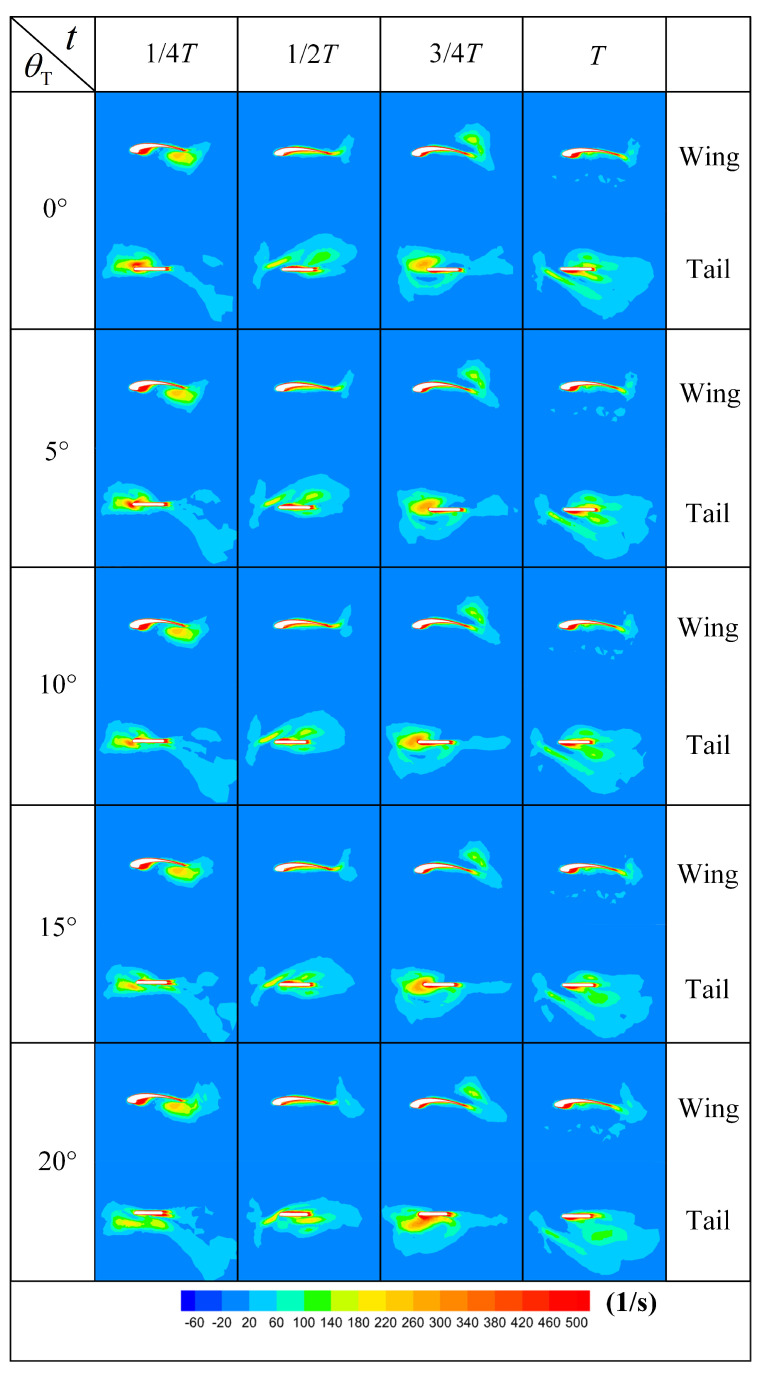
Vorticity contours at 0.1a cross-section under varying tail twist angles.

Figure 15 presents the flow field vorticity contours around the wing and tail at different instants over a single flapping cycle at the 0.1a cross-section for varying tail twist angles. As seen, the vortex structures on the upper and lower surfaces of the tail vary at different time instants during the flapping cycle. This arises because the tail is positioned adjacent to the wing trailing edge on the flapping-wing aircraft and is therefore subjected to the downwash flow field generated by the wing flapping across different phases, which drives the changes in the vortex structures within the tail region.

Meanwhile, as the tail twist angle $\theta_{T}$ increases, the tail profile at the 0.1a cross-section gradually shifts upward. At the same instant, the tail cross-section lies in distinct regions of the wing-induced downwash flow field for varying tail twist angles $\theta_{T}$. At *t* = 1/4 *T*, the peak vorticity magnitude is concentrated at the leading edge of the tail’s upper surface for a 0° tail twist angle $\theta_{T}$. As the tail twist angle rises, the tail cross-section passes upward through the vortex structures shed from the wing and progressively moves away from the wing’s downwash flow field. Accordingly, the location of the tail’s peak vorticity shifts downward from the upper to the lower surface, accompanied by a gradual reduction in the strength of the vortex structures.

**Figure 16:**
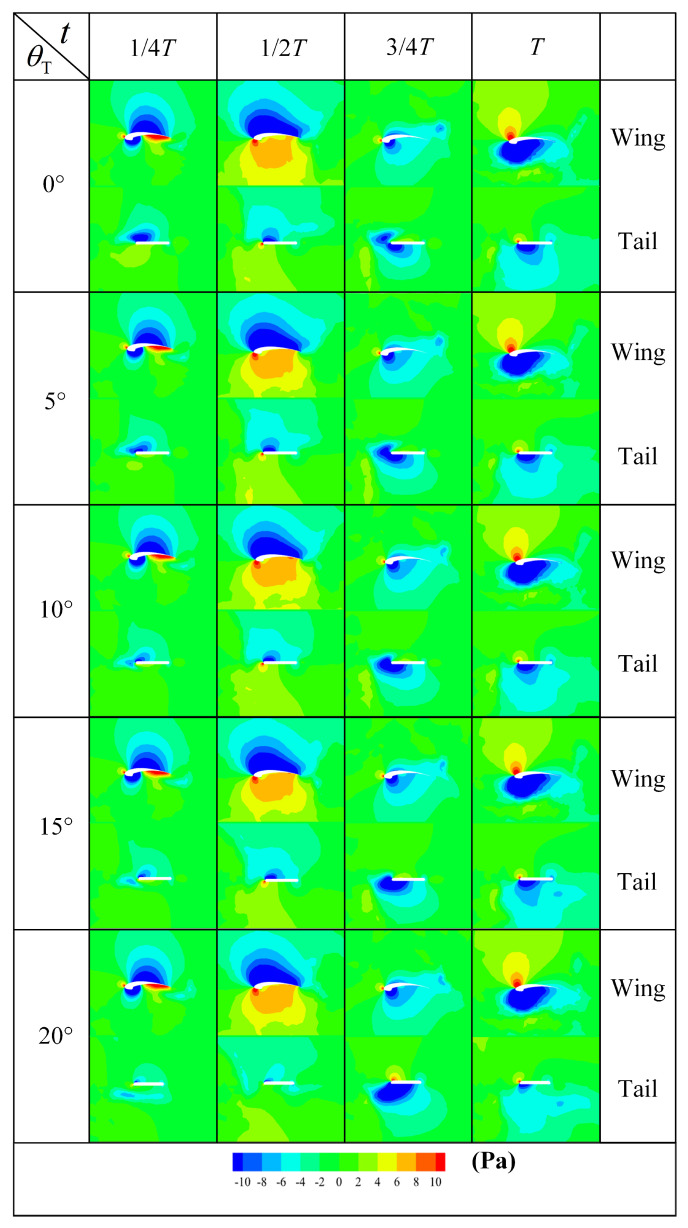
Pressure contours at 0.1a cross-section under varying tail twist angles.

In conjunction with the pressure contours at the 0.1a cross-section in Figure 16, our analysis reveals that, consistent with the changes in strength and location of the vortex structures observed in Figure 15, the high-strength vortex structure at the leading edge of the tail’s upper surface at *t* = 1/4 *T* (for a tail twist angle $\theta_{T}$ = 0°) corresponds directly to the low-pressure region at the same location in Figure 16. As the tail twist angle $\theta_{T}$ increases, the low-pressure region shifts progressively downward, with a concurrent reduction in its magnitude. This finding demonstrates that the downwash flow field induced by the wing across different flapping phases modulates the tail’s vortex structures, with this effect varying with changes in the tail position. This in turn modifies the pressure difference between the tail’s upper and lower surfaces and ultimately drives the changes in the overall moments of the full bird-inspired FWAV.

**Figure 17:**
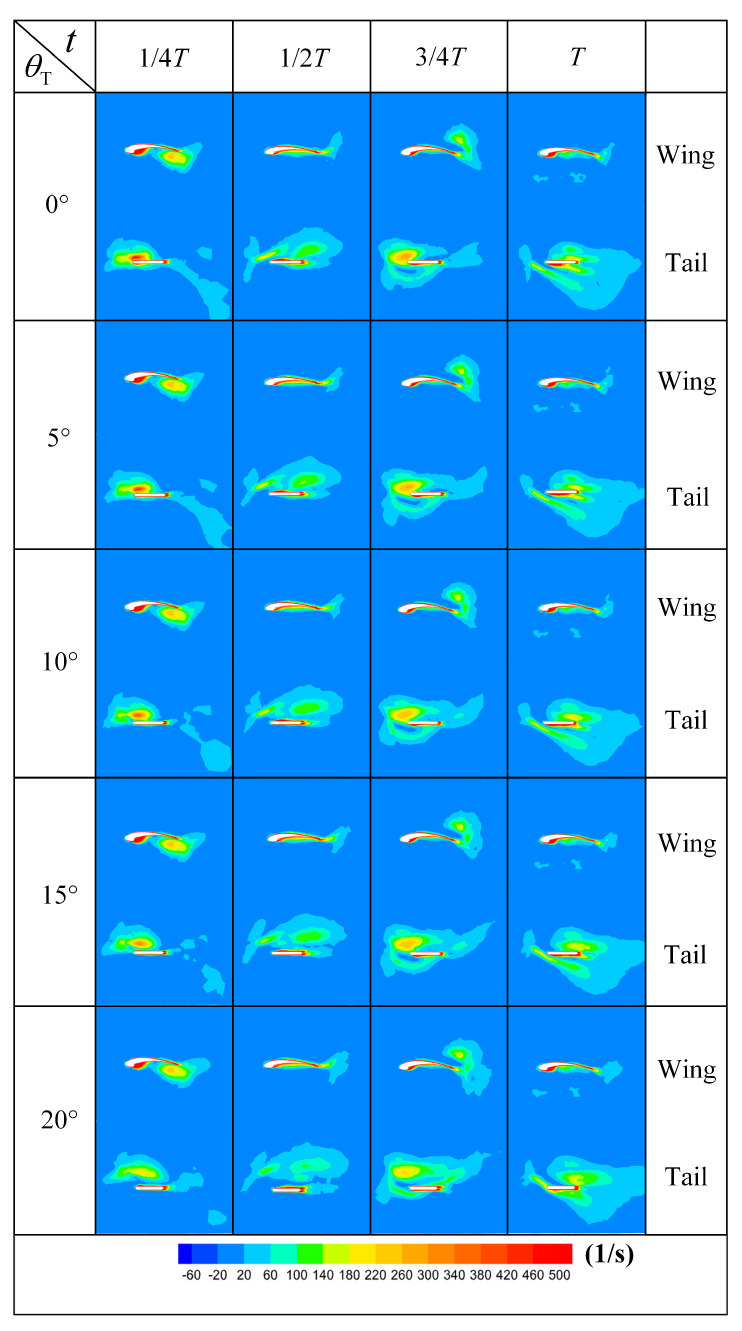
Vorticity contours at −0.1a cross-section under varying tail twist angles.

To further elucidate how the tail twist angle modulates the variation in the full vehicle’s overall moments, we perform an analysis combined with the flow field vorticity and pressure contours at the −0.1a cross-section. As shown in Figure 17, the tail profile at the −0.1a cross-section shifts progressively downward as the tail twist angle $\theta_{T}$ increases. At *t* = 1/4 *T*, the tail cross-section moves downward away from the vortex structures shed from the wing, accompanied by a gradual reduction in the strength of the vortex structures on the tail’s upper surface.

**Figure 18:**
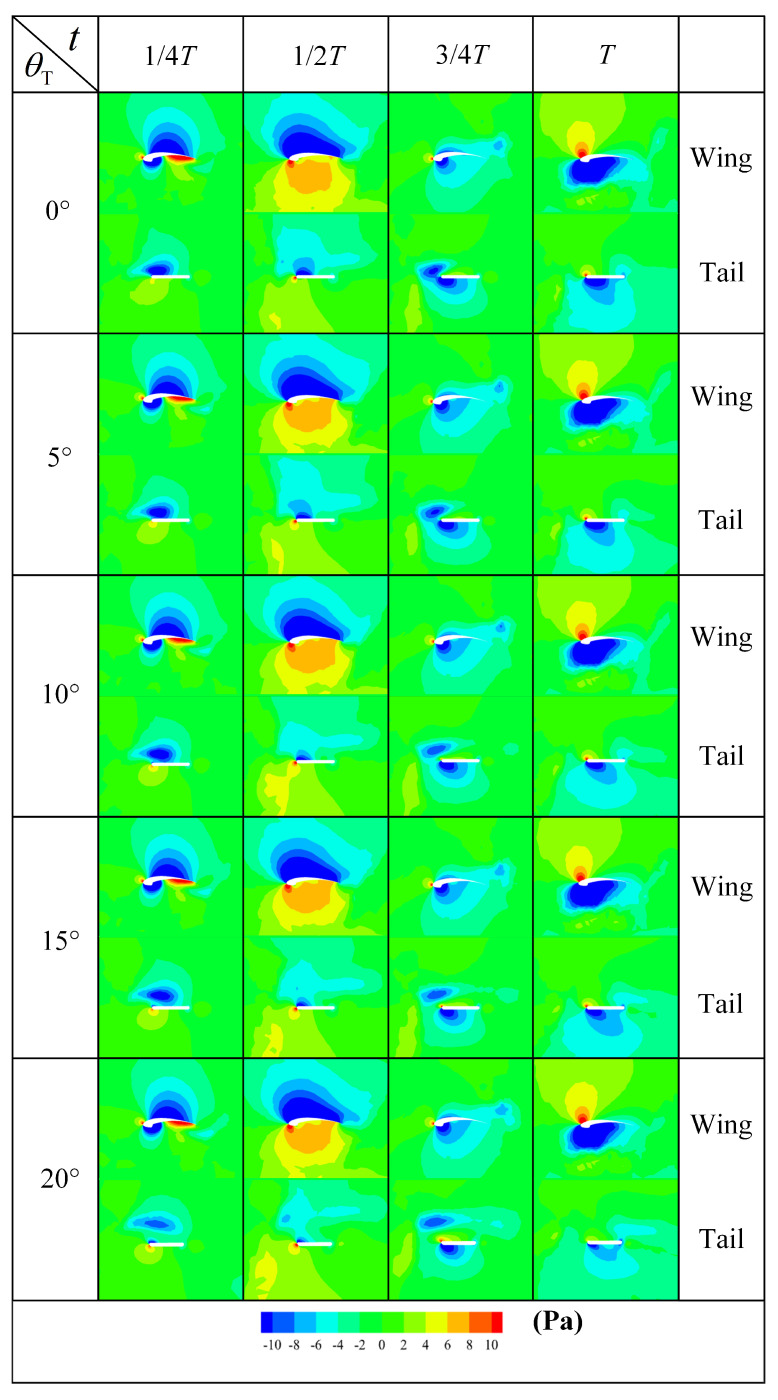
Pressure contours at −0.1a cross-section under varying tail twist angles.

Meanwhile, analysis of the pressure contours at the −0.1a cross-section for varying tail twist angles $\theta_{T}$ in Figure 18 shows that at *t* = 1/4 *T*, as the tail profile shifts downward, the low-pressure region induced by wing vortex shedding moves progressively away from the tail’s upper surface, with a concurrent reduction in its magnitude. In contrast, the magnitude of the high-pressure region on the lower surface of the tail’s leading edge increases gradually.

Through the combined analysis of the vorticity and pressure contours at the 0.1a and −0.1a cross-sections, it was clearly observed that as the tail twist angle increased, the left and right tails were affected by the wing downwash flow at different positions. Furthermore, at 1/4 *T* when the tail twist angle $\theta_{T}$ increased to 20°, it could be seen from the vorticity and pressure contours at the 0.1a and −0.1a cross-sections that the low-pressure region on the upper surface of the tail weakened at 20°, which corresponded to the decrease in the instantaneous pitching moment at 1/4 *T* in Figure 11a.

Meanwhile, between 1/2 *T* and 3/4 *T*, the low-pressure regions at the 0.1a and −0.1a tail cross-sections gradually expanded, indicating that the pressure difference between the upper and lower surfaces of the tail exhibited a trend of first decreasing and then increasing in this interval, which corresponded to the trough in the instantaneous pitching moment curve between 1/2 *T* and 3/4 *T* in Figure 11a. In addition, the tail twist generated pressure differences in opposite directions between the upper and lower surfaces of the left and right tails, which in turn induced rolling and yawing moments opposite to the tail twist direction.

### 3.2. Effect of Tail Pitch Angle During Climbing Flight Phase on Aerodynamics

**Figure 19:**
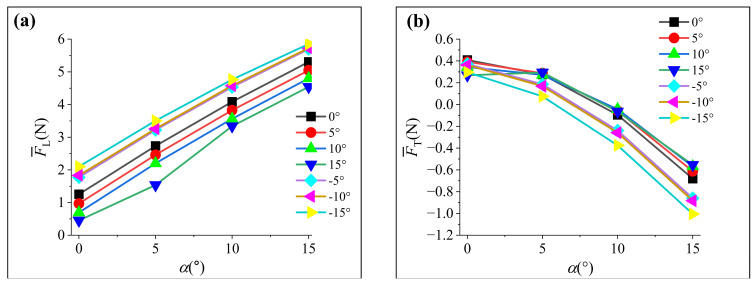
Line plots of cycle-averaged lift and thrust over a single flapping cycle under different full-vehicle angles of attack and tail pitch angles. (**a**) Line plot of cycle-averaged lift $\bar{F_{L}}$. (**b**) Line plot of cycle-averaged thrust $\bar{F_{T}}$.

Based on the aircraft attitude changes during the climbing phase in Section 2.4 and the motion parameter settings in Table 6, we conduct numerical simulations. Figure 19a,b show the line plots of the cycle-averaged lift $\bar{F_{L}}$ and cycle-averaged thrust $\bar{F_{T}}$ of the bird-inspired flapping-wing aircraft over a single flapping cycle for varying full-vehicle angles of attack and tail pitch angles, respectively.

From the cycle-averaged lift $\bar{F_{L}}$ line plot in Figure 19a, it is evident that for a fixed tail pitch angle $\beta_{T}$, the cycle-averaged lift $\bar{F_{T}}$ of the bird-inspired FWAV increases monotonically with the full-vehicle angle of attack α. For a fixed full-vehicle angle of attack α, the magnitude of $\bar{F_{L}}$ is modulated by both upward ($\beta_{T}$ > 0°) and downward ($\beta_{T}$ < 0°) tail deflections: $\bar{F_{L}}$ decreases progressively with increasing $\beta_{T}$ for upward-deflected tails and increases progressively with decreasing $\beta_{T}$ for downward-deflected tails.

The cycle-averaged thrust $\bar{F_{T}}$ line plot in Figure 19b shows that $\bar{F_{T}}$ decreases with increasing full-vehicle angle of attack α for all tail pitch angles $\beta_{T}$, with the only exception of $\beta_{T}$ = 15°, where $\bar{F_{T}}$ increases as α rises from 0° to 5°. For α > 5°, $\bar{F_{T}}$ increases with rising $\beta_{T}$ when $\beta_{T}$ > 0°, and decreases with reducing $\beta_{T}$ when $\beta_{T}$ < 0°.

The results in Figure 19 confirm that the impact of tail pitch angle $\beta_{T}$ on full-vehicle lift and thrust is dependent on the full-vehicle angle of attack α. Furthermore, upward and downward tail deflections drive opposite trends in the variation in full-vehicle lift and thrust.

**Figure 20:**
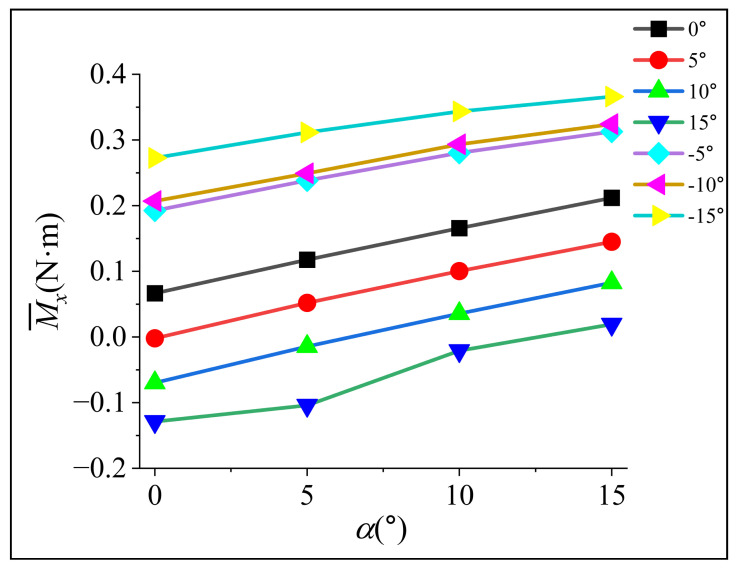
Line plots of cycle-averaged pitching moment under varying full-vehicle angles of attack and tail pitch angles.

To further investigate how tail pitch angle $\beta_{T}$ affects the full-vehicle pitching attitude under varying full-vehicle angles of attack α, we analyze the cycle-averaged full-vehicle moment $\bar{M_{x}}$ over a single flapping cycle. Figure 20 shows the line plot of $\bar{M_{x}}$ variation for different tail pitch angles $\beta_{T}$ across varying full-vehicle angles of attack α.

As can be seen from Figure 20 above, at a constant full-vehicle angle of attack α, when the tail pitch angle $\beta_{T}$ = 0° or 5°, the cycle-averaged full-vehicle pitching moment $\bar{M_{x}}$ over a single flapping cycle decreases with the increase in $\beta_{T}$. However, for all tested full-vehicle angles of attack α, the cycle-averaged pitching moment $\bar{M_{x}}$ over a single motion cycle is greater than 0. That is, the upward deflection of the tail at this time reduces the nose-up pitch degree of the vehicle, and the nose-down pitching moment increases with the rise in the full-vehicle angle of attack α. When the tail pitch angle $\beta_{T}$ is 10° or 15° and the full-vehicle angle of attack α is 0° or 5°, the cycle-averaged pitching moment $\bar{M_{x}}$ over a single cycle is less than 0. At this point, the upward deflection of the tail induces nose-up pitching of the vehicle, and the nose-up pitching moment increases with the increase in $\beta_{T}$. When the full-vehicle angle of attack α is 10° or 15°, the direction of the cycle-averaged pitching moment $\bar{M_{x}}$ at $\beta_{T}$ = 10° reverses, switching from a nose-up pitching moment to a nose-down pitching moment.

In addition, when the tail pitch angle $\beta_{T}$ < 0°, the cycle-averaged pitching moment over a single cycle is consistently greater than 0. That is, the downward deflection of the tail always generates a moment that drives the nose of the vehicle to pitch down, and the magnitude of the moment increases with the rise in both the tail pitch angle $\beta_{T}$ and the full-vehicle angle of attack α.

The data in Figure 20 indicate that when only the full-vehicle angle of attack α of the vehicle is adjusted ($\beta_{T}$ = 0°), the cycle-averaged pitching moment $\bar{M_{x}}$ of the flapping-wing aircraft over a single cycle is greater than 0, which causes nose-down pitching of the vehicle and makes it difficult to maintain the preset angle of attack to complete the flight in the climbing phase. Therefore, during the climbing phase, it is necessary to appropriately adjust the upward deflection angle of the vehicle’s tail to maintain a proper full-vehicle angle of attack. Meanwhile, the nose-down pitching moment generated by the downward deflection of the tail can be combined to stabilize the longitudinal moment, ensuring that the vehicle maintains dynamic balance.

**Figure 21:**
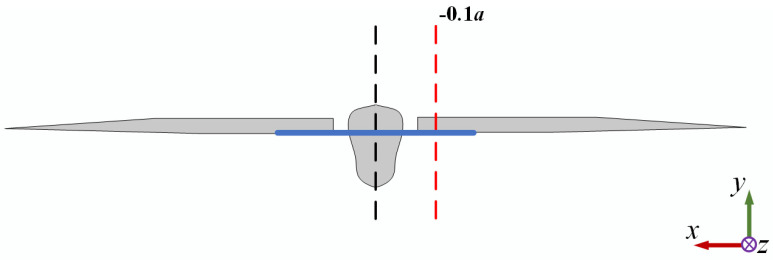
Schematic of cross-section location.

To further elucidate how the combined action of tail pitch angle and full-vehicle angle of attack α modulates the flow field structure of the bird-inspired flapping-wing aircraft, we analyze the flow field vorticity and pressure contours for full-vehicle angles of attack α of 0° at tail pitch angles $\beta_{T}$ of 10°, 0° and −10°, to characterize the aerodynamic coupling between the two parameters. As presented in Figure 21, the analysis is performed on the −0.1a cross-section on the right side, which is symmetric about the fuselage central axis.

**Figure 22:**
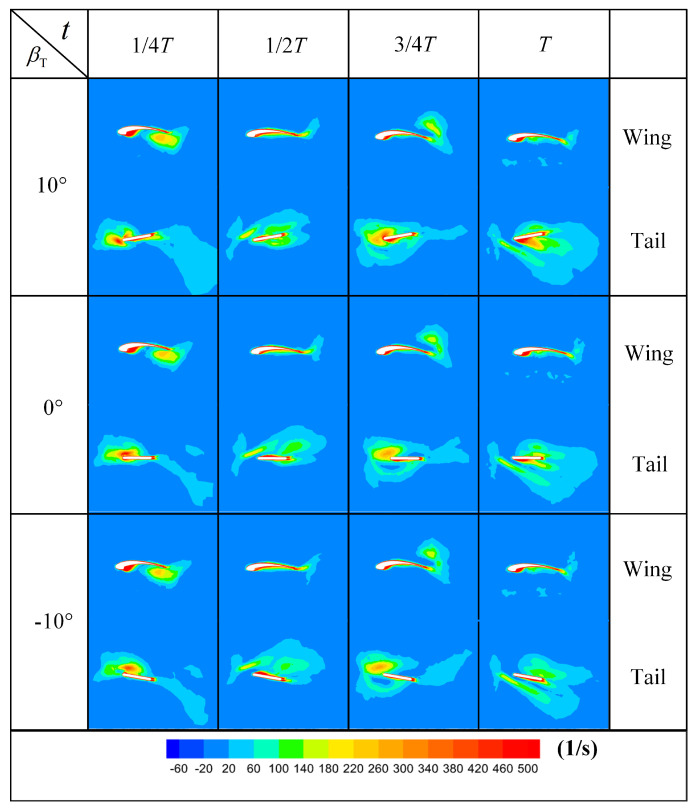
Vorticity contours (α = 0°; $\beta_{T}$ = 10°, 0°, −10°).

The corresponding flow field vorticity contours are presented in Figure 22. It is evident that, for a fixed full-vehicle angle of attack, varying tail pitch angles $\beta_{T}$ modify the region where the wing-shed vortex structures interact with the tail. At the instant of *t* = 1/4 *T*, for $\beta_{T}$ = 0°, the vortex structures shed from the wing surface are concentrated at the front end of the tail’s upper surface. For $\beta_{T}$ = 10°, the tail position impedes the backward convection of the wing-shed vortex structures, confining them to the leading edge of the tail, resulting in a reduced influence area but increased vortex strength. For $\beta_{T}$ = −10°, the tail position imposes less obstruction on the shedding and convection of the wing-shed vortex structures, which then convect backward along the tail’s upper surface, leading to an increased influence area but reduced vortex strength.

**Figure 23:**
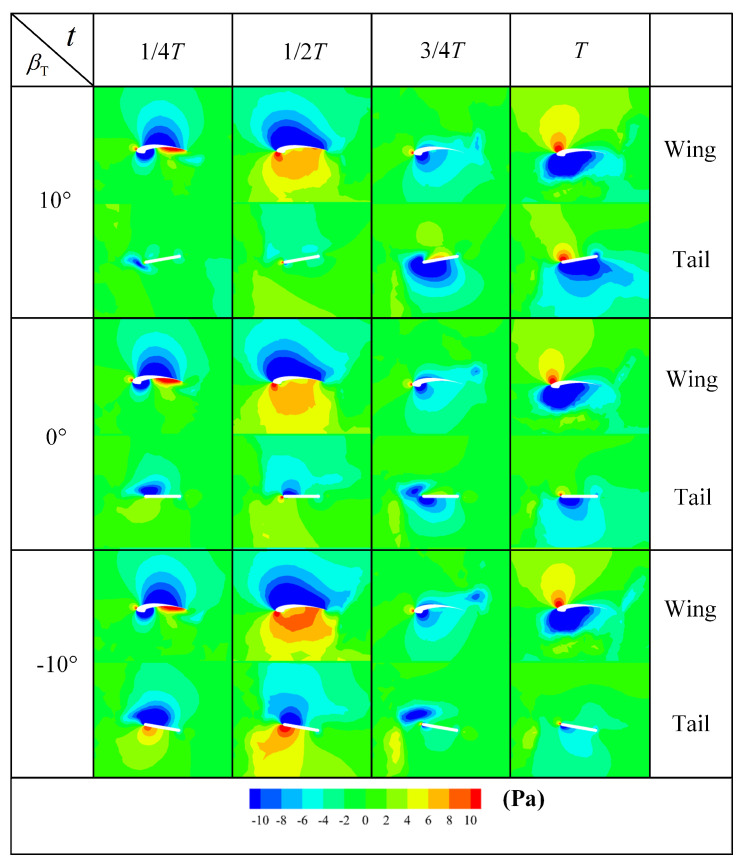
Pressure contours (α = 0°; $\beta_{T}$ = 10°, 0°, −10°).

Analysis of the pressure contours in Figure 23 at α = 0° for $\beta_{T}$ = 10°, 0°, −10° shows that the low-pressure region location matches the vortex structure concentration observed in Figure 22. At *t* = 1/4 *T*, the low-pressure region is concentrated at the front of the tail’s upper surface for $\beta_{T}$ = 0°; at the tail’s leading edge with a reduced area for $\beta_{T}$ = 10°; and spread over the tail’s upper surface for $\beta_{T}$ = −10°, where the upper-to-lower surface pressure difference increases significantly. This additional lift generated by the tail induces a full-vehicle nose-down pitching moment.

**Figure 24:**
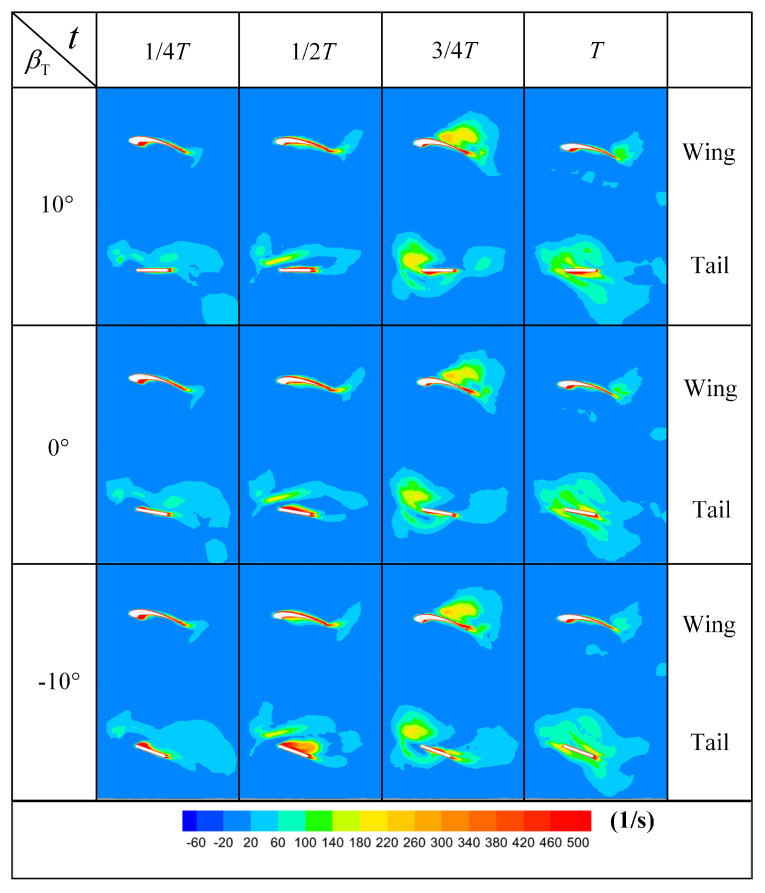
Vorticity contours (α = 10°; $\beta_{T}$ = 10°, 0°, −10°).

Furthermore, by adjusting the full-vehicle angle of attack, the effect of changing the angle of attack on the flow field structure is examined for the same tail pitch angle $\beta_{T}$. Figure 24 below presents the flow field vorticity contours at the full-vehicle angle of attack α = 10° with tail pitch angles $\beta_{T}$ = 10°, 0°, and −10°.

As can be seen from Figure 24, the change in the full-vehicle angle of attack alters the influence region of the wing downwash flow and the path of the vortices shed from the wing surface. At the instant of *t* = 1/2 *T*, when $\beta_{T}$ = −10°, the tail position is closer to the downstream path of the vortices shed from the wing surface, causing the shed vortex structures to stagnate on the tail’s upper surface. Meanwhile, influenced by the tail’s upper surface, the strength of the stagnant vortex structures increases. When $\beta_{T}$ = 10°, the tail position is separated from the main convective path of the wing-shed vortices, resulting in a weak influence of the tail on the vortex structures.

**Figure 25:**
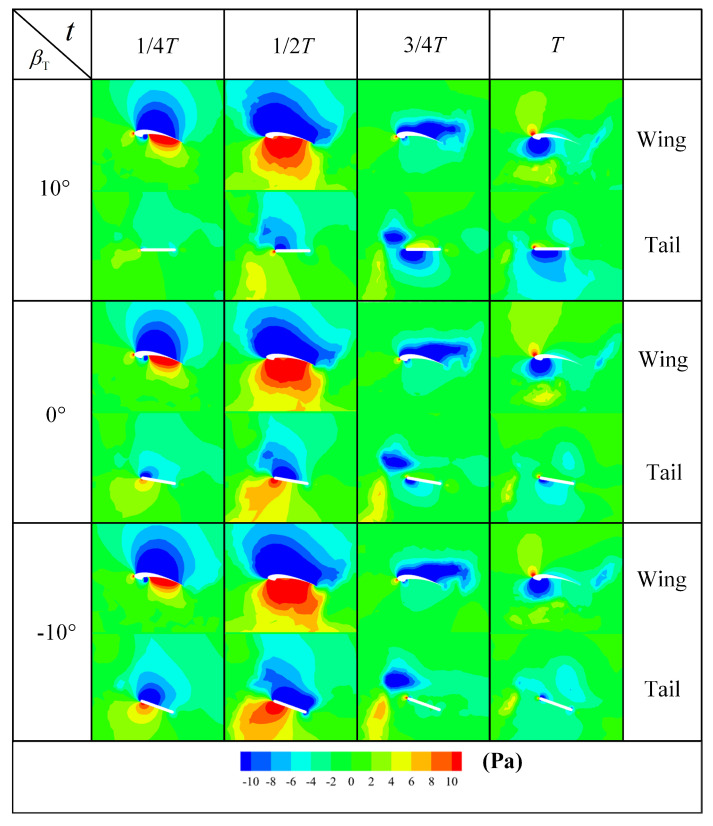
Pressure contours (α = 10°; $\beta_{T}$ = 10°, 0°, −10°).

Meanwhile, we analyze the pressure contours in Figure 25 for α = 10° at $\beta_{T}$ = 10°, 0°, and −10°. At *t* = 1/2 *T*, the maximum overlap between the tail- and wing-shed vortices occurs at $\beta_{T}$ = −10°, where the low-pressure core of the vortices directly impinges on the tail surface, creating a pronounced pressure difference and the largest aerodynamic load fluctuation on the tail. For $\beta_{T}$ = 10°, by contrast, the tail has minimal overlap with the vortex path, and the vortex structures exert only a weak pressure induction effect on the tail surface, leading to a more uniform pressure distribution and milder load fluctuations.

Combined with Figure 22, Figure 23, Figure 24 and Figure 25 above, it can be observed that the angle of attack α and the tail pitch angle $\beta_{T}$ exhibit a mutual coupling effect during the climbing phase. This aerodynamic coupling effect manifests as follows: when the flapping-wing aircraft operates at different angles of attack α, it induces changes in the downwash intensity and wake vortex trajectory generated by the flapping motion of the wings. These changes exert distinct effects on the upper and lower surfaces of the tail region. Furthermore, there exists an optimal interval for the aerodynamic influence of the wings on the tail; when the attitudes of the wings and tail fall within this interval, they yield different beneficial effects on the aircraft.

A representative case of this optimal coupling interval is illustrated in Figure 19b, revealing that when the angle of attack α ranges from 0° to 5° and the tail pitch angle $\beta_{T}$ = 5°, the leading edge of the tail precisely overlaps with the high-velocity region below the trailing edge vortex shed during the downstroke phase of the wings (TEV-down). As the vortex core passes over the lower surface of the tail, it generates an additional downward induced velocity, thereby increasing the effective angle of attack of the tail. Simultaneously, the wing-shed vortex undergoes favorable interaction with the attached flow over the tail: the vortex core is stretched and accelerated by the tail surface, and its rotational kinetic energy is converted into forward thrust of the aircraft via induced lift, further enhancing the overall average thrust of the aircraft.

These findings not only confirm the existence of the optimal coupling interval but also demonstrate that the aerodynamic characteristics of the flapping-wing aircraft are governed by the mutual coupling between the full-vehicle angle of attack and the tail pitch angle. Beyond thrust performance, this coupling effect also significantly affects the pitching moment characteristics of the aircraft. During flight, the vehicle can switch between stable and unstable flight states by adjusting the tail pitch angle. For example, downward deflection of the tail can enhance the influence of wing-shed vortices on the pitching moment to improve maneuver response, while upward deflection of the tail can weaken this influence to enhance flight stability.

## 4. Conclusions

In this work, we established a three-dimensional numerical flow field simulation model based on a simplified 3D geometry of a bird-inspired flapping-wing aircraft and performed numerical simulations using the overset mesh method. We systematically investigated the effects of kinematic and attitude parameter variations on the aerodynamic performance and flow field structure of the flapping-wing aircraft in two typical flight phases: level flight and climbing. The key conclusions are summarized as follows:

(1) During the level flight phase, the tail twist angle has a negligible effect on lift and thrust (with a peak variation of no more than 0.2 N), but significantly modulates the pitching, rolling and yawing moments. The optimal tail twist angle is 0°~15°, at which the steering moments are linearly controllable without the risk of abrupt attitude changes; when the twist angle exceeds 20°, the pitching moment increases abruptly by 6%, which may lead to a loss of flight altitude.

(2) During the climbing phase, for every 5° increase in the full-vehicle angle of attack, the cycle-averaged lift increases by approximately 1.2 N, while the cycle-averaged thrust decreases by approximately 0.15 N. At a full-vehicle angle of attack α= 15°, the optimal tail pitch angle is 5°, at which the cycle-averaged pitching moment of the full vehicle is nearly zero, enabling longitudinal stability to be maintained while retaining the maximum lift; downward deflection of the tail ($\beta_{T}$ < 0°) amplifies the nose-down pitching moment and is detrimental to climbing performance.

Unlike previous studies that treated the wing and tail as independent aerodynamic surfaces and focused primarily on their aerodynamic interaction during the gliding phase, this study systematically revealed the effects of key kinematic parameters on the aerodynamic performance of flapping-wing aircraft across distinct flight phases, as well as the underlying flow field mechanisms.

The results obtained in this study can provide guidance for the attitude control of aircraft across different flight phases. Specifically, they allow the establishment of a more precise mapping relationship between tail twist, pitch angle and full-aircraft aerodynamic moments, which in turn enables coordinated adjustment of multiple control parameters to achieve more stable turning or climbing maneuvers. These findings also provide a robust theoretical basis for the parameter optimization and attitude control system design of avian-inspired flapping-wing aircraft.

Although this paper further reveals the effects of different flight phases on the aerodynamic performance of flapping-wing aircraft and the underlying flow field mechanisms, it has certain limitations. Further optimization studies can be conducted in the following aspects:

(1) Model preprocessing: In this study, the tail was simplified as a rigid fan-shaped thin plate and the wings as rigid wings, ignoring the flexible deformation and feather gap effects of real birds. This may cause numerical deviations between the aerodynamic results and the actual situation. Subsequent research can establish a fluid–structure interaction model of the flexible tail to investigate the influence of tail flexibility on the wing–tail coupling effect.

(2) Flight phase coverage: This study only analyzed two typical flight phases, level flight and climbing, and did not address complex maneuvering states such as diving and hovering.

(3) Flow field conditions: This study was conducted under a fixed incoming flow velocity and constant flapping frequency, without considering the impacts of complex environments such as atmospheric turbulence and gusts. Subsequent research can introduce advanced atmospheric turbulence models to analyze how the coordinated motion of wings and tails can improve the aerodynamic stability of flapping-wing aircraft under complex flow field conditions.

## Figures and Tables

**Table 1:** Basic parameters of the FWAV three-dimensional simulation model.

Full Vehicle Overall Length [m]	Total Bilateral Wingspan [m]	Area of the Tail Assembly [$m^{2}$]	Aspect Ratio
0.64	1.2	0.05	6.55

**Table 2:** Geometric dimension parameters of each computational domain.

Background Mesh [m]	Fuselage Foreground Mesh [m]	Inner Wing Foreground Mesh [m]	Outside Wing Foreground Mesh [m]	Tail Assembly Foreground Mesh [m]
120×12×8	∅0.7×2	∅0.7×2	∅0.6×2	∅0.6×1.3

**Table 3:** Verification model parameters and their motion parameters.

Wingspan [m]	Chord Length [m]	Frequency [Hz]	Maximum Flapping Angle [°]	Inflow Velocity [m/s]	Angle of Attack [°]
0.4	0.08	8	38.44	8	7

**Table 4:** Simulation parameter settings for mesh and time-step independence verification.

Inflow Velocity [m/s]	Angle of Attack [°]	Frequency [Hz]	Maximum Flapping Upward Angle [°]	Maximum Flapping Downward Angle [°]
5	0	5	38	20

**Table 5:** Different mesh densities.

Mesh Densities	Total Foreground Mesh Count	Background Mesh Count
Coarse	$1.78\times 10^{6}$	$5.02\times 10^{5}$
Medium	$1.78\times 10^{6}$	$9.65\times 10^{5}$
Fine	$1.78\times 10^{6}$	$1.9\times 10^{6}$

**Table 6:** Kinematic parameters and simulation cases.

Inflow Velocity [m/s]	Tail Twist Angle [°]	Frequency [Hz]	Maximum Flapping Upward Angle [°]	Maximum Flapping Downward Angle [°]
5	0	5	38	20

## Data Availability

The original contributions presented in this study are included in the article. Further inquiries can be directed to the corresponding authors.
